# Investigation of Plasticity Effects on Growing Fatigue Cracks Using the CJP Model of Crack Tip Fields

**DOI:** 10.3390/ma16175744

**Published:** 2023-08-22

**Authors:** José Manuel Vasco-Olmo, Alonso Camacho-Reyes, Giancarlo Luis Gómez Gonzales, Francisco Díaz

**Affiliations:** Departamento de Ingeniería Mecánica y Minera, University of Jaén, 23071 Jaén, Spain; acreyes@ujaen.es (A.C.-R.); glgomez@ujaen.es (G.L.G.G.); fdiaz@ujaen.es (F.D.)

**Keywords:** fatigue crack growth, crack tip fields, crack shielding, CJP model, digital image correlation (DIC)

## Abstract

A growing fatigue crack gives rise to a plastic enclave that envelops the crack and can exert a shielding effect on the crack from the global elastic stress field driving fatigue propagation. This work presents the potential of the CJP model of crack tip fields to investigate the plasticity-induced shielding effects on growing fatigue cracks as well as its ability to characterise the size and shape of the plastic zone generated at the tip of a growing fatigue crack. The model was specifically developed to consider the influence of the plastic enclave generated around a fatigue crack on the surrounding elastic material. Different aspects related to fracture mechanics and its implications for fatigue crack growth have been investigated, namely plasticity-induced crack shielding, the retardation effect induced on fatigue crack growth due to the application of an overload and the estimate of the size and shape of the crack tip plastic zone. The model has been successfully applied by analysing displacement fields experimentally measured by DIC in different CT specimens made of 2024-T3 aluminium alloy and commercially pure titanium. Results presented in this work intend to contribute to a better understanding of the shielding effects during fatigue crack growth.

## 1. Introduction

During fatigue crack growth, a plastic zone at the crack tip is generated and a region of permanent deformation (known as plastic wake) is left along the crack flanks as the crack propagates. This plastic enclave will shield the crack from the influence of the elastic stress field driving fatigue crack propagation, giving rise to premature contact between the crack faces during unloading. This contact effect is known as plasticity-induced crack closure [1] and it induces a retardation effect on the fatigue crack growth rates. In present times, crack closure is recognised as one of the fatigue crack shielding mechanisms that lead to a reduction in the effective range of stress intensity factors driving fatigue crack propagation [2].

The stress intensity factor (SIF) is one of the most relevant and commonly used parameters to characterise the stress and displacement fields around a crack tip. In addition, under small-scale yielding conditions, the parameter used to characterise fatigue crack growth is the stress intensity factor range (Δ*K*) through the so-called Paris ‘law’ [3]. Therefore, the calculation of the stress intensity factor and its subsequent analysis can be a very useful way to explore the shielding effects on the applied elastic field and the retardation effect that can be induced on fatigue crack propagation. Now, the use of full-field optical techniques [4] has become very popular for the study of structural integrity and material durability problems, such as thermoelastic stress analysis (TSA) [5], digital photoelasticity, electronic speckle pattern interferometry (ESPI) [6] or Digital Image Correlation (DIC) [7,8]. Among them, DIC [9] is an optical technique performed to measure displacements and strains in mechanical components and currently it is widely used for the study of many applications due to its easy implementation. At present, DIC is a well-established optical technique for the investigation of different phenomena observed in fracture mechanics and fatigue crack propagation. In this work, DIC is used to experimentally evaluate different problems related to the characterisation of fatigue crack growth, such as the crack shielding effect, the effects due to the application of overloads and the estimate of the crack tip plastic zone.

In the literature, there are different models based on linear elastic fracture mechanics (LEFM) to describe the stress and displacement fields near a crack tip, including the model based on Westergaard’s equations [10], the model based on Williams’ expansion series [11], the model based on Muskhelishvili’s complex potentials [12] and the CJP model [13]. These models have been described in terms of stresses (e.g., Westergaard [10], Williams [14], Muskhelisvili [15], and CJP [16]) and displacements (e.g., Westergaard [10], Williams [17], Muskhelishvili [18], and CJP [16]). The first three models do not consider any effects of plasticity generated in a growing fatigue crack. However, the CJP model has been developed to consider the influence of the plastic enclave generated around a fatigue crack by analysing its shielding effects on the surrounding elastic stress field. Since the objective of the present paper is to investigate the effects of plasticity on propagating fatigue cracks, the CJP model has been used to characterise the stress and displacement fields around the tip of fatigue cracks.

It is recognised in the fracture mechanics and fatigue community that the simultaneous quantification of the size and shape of the plastic zone generated at the tip of propagating fatigue cracks is a complex problem. In the literature, there are many research works on the numerical and experimental quantification of the crack tip plastic zone during fatigue crack propagation. The techniques for the experimental quantification include microhardness measurements, etching, optical interference, microstrain gauges, and electron microscopy [19]. More modern experimental techniques have been applied to quantitatively obtain plastic strain fields on growing fatigue cracks and hence for the crack tip plastic zone estimate; these techniques include synchrotron X-ray diffraction [20], DIC [21], thermoelastic stress analysis (TSA) [22], and electron backscatter diffraction (EBSD) [23]. For example, Carrera et al. [20] have used synchrotron X-ray diffraction on bainitic steel by applying Von Mises yield criterion to generate the equivalent strain map from the elastic strain fields. Zhang and Liu [21] combined DIC results with the material constitutive relationship to obtain the plastic zone size at the tip of growing fatigue cracks. Patki et al. [22] used TSA to measure the size of the crack tip plastic zone in 2024 aluminium alloy using a description of the stress fields at the crack tip based on Muskhelishvili’s complex potentials. The authors developed a novel methodology based on the difference between the applied load and the TSA signal for the direct measurement of the extent of the plastic zone generated at the crack tip. Finally, Yang et al. [23] defined a new method to find the extent of plastic strain based on applying a discrete Fourier transform (DFT) to images obtained by EBSD.

The present work combines the use of DIC to measure the experimental displacement fields on growing fatigue cracks in different CT specimens made of 2024-T3 aluminium alloy and commercially pure titanium together with the use of the CJP model for the characterisation of the crack tip displacement fields. The model has been successfully applied to the analysis of different phenomena related to the fatigue crack growth characterisation, including the shielding effect induced by the plastic enclave surrounding a fatigue crack, the retardation effect on the crack growth rates induced by the application of single overloads and the estimate of the size and shape of the plastic zone generated at the crack tip. Results show the ability of the CJP model to experimentally evaluate phenomena where plasticity plays a relevant role in fatigue crack growth through its shielding effects from the influence of the elastic stress field as a driving mechanism. It is believed that this work can contribute to a better understanding of the mechanisms that control fatigue crack propagation.

## 2. Background of the CJP Model

In the original work reported by James et al. [24], transmission photoelasticity was combined with a mathematical analysis for crack tip stresses that incorporated wake contact forces to explore the role of crack closure on fatigue crack growth. Polycarbonate compact tension (CT) specimens containing growing fatigue cracks were used to analyse the variation in the wake contact forces through the loading cycle. The authors proposed from their results that the plastic enclave generated around propagating fatigue cracks induces shielding effects on the crack from the influence of the surrounding elastic stress field. Further investigation of this first mathematical analysis including an assessment of the shielding mechanisms during fatigue crack growth was reported by Pacey et al. [25]. The authors developed a simplistic photoelastic model based on the Muskhelishvilian approach [12], which demonstrated that highly localised stress effects from plasticity-induced shielding could be captured by the global photoelastic stress field. The model was fitted to full-field experimental photoelastic data on polycarbonate CT specimens, which is ductile enough to allow the growth of a fatigue crack [24]. The stress intensity factors during two load cycles were calculated, showing evidence of crack closure through the measurement of the crack opening and closing loads. These two previous works constituted the germ of what is currently known as the CJP model [13], which uses the complex potentials of Muskhelishvili but does not employ the conformal mapping utilises by Pacey et al. [25]. The following paragraphs describe the current conception of the CJP model.

**Figure 1:**
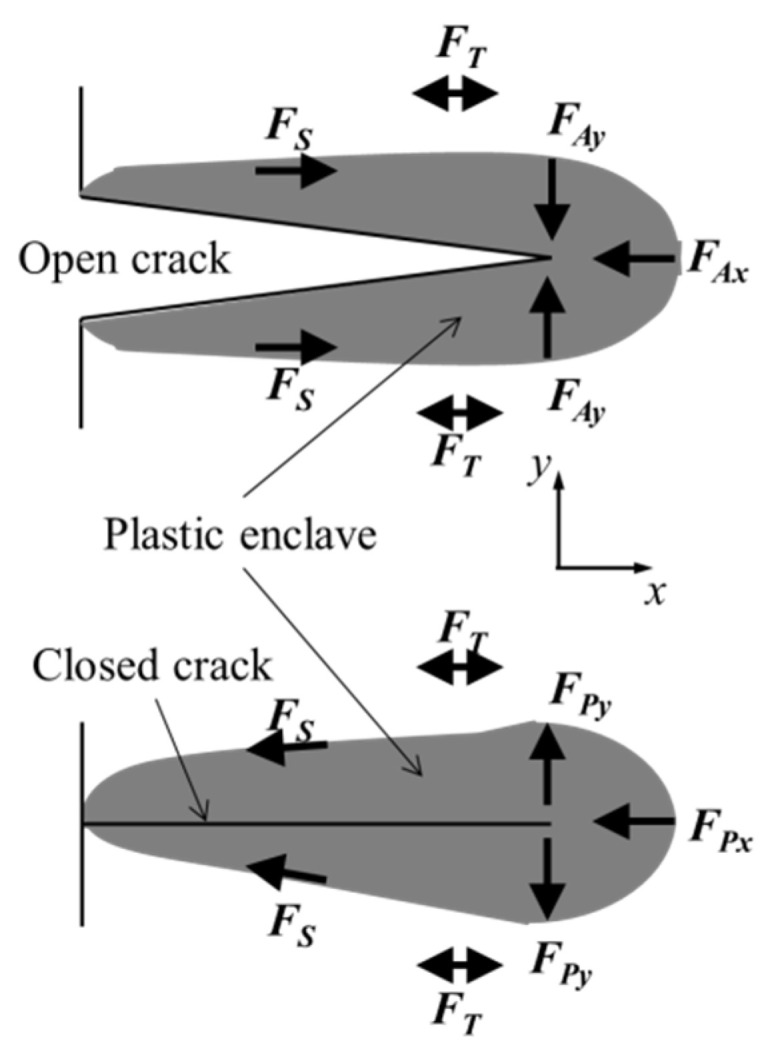
Schematic diagram illustrating the forces acting on the elastic field surrounding the plastic enclave around an open (**top**) and closed (**bottom**) growing crack.

The CJP model was proposed and developed by Christopher, James, and Patterson [13], being referred to as this by its originators. It is a mathematical model to characterise the stress and displacement fields around the tip of a fatigue crack. It was developed to explicitly consider the influences of the plastic enclave surrounding a growing fatigue crack on the applied elastic field. The authors postulated that this embedded plastic region around the crack will shield it from the full influence of the applied stress field that drives fatigue crack growth. This crack shielding includes the effect of the contact forces between the crack flanks due to crack closure as well as the effect of shear stresses induced by compatibility between the plastic enclave and the surrounding elastic stress field. The model characterises therefore not only the conditions at the crack tip but also the conditions behind the tip since the crack propagation rates are influenced by the conditions ahead and behind the crack tip. Figure 1 illustrates schematically the forces acting on the elastic stress field surrounding the plastic enclave generated during fatigue crack growth. This illustration is similar to the schematic diagram reported by the authors in their original publication of the CJP model [13].

These forces include [13]: *F_Ax_* and *F_Ay_* are the reaction forces to the applied remote load. *F_T_* is the force induced by the T-stress, which is also generated by the remote applied load and whose sense is dependent on the specimen geometry. The above forces generate a plastic zone ahead of the crack tip such that the material will become permanently deformed inducing during unloading forces *F_Px_* and *F_Py_*. *F_Py_* is extensive in the perpendicular direction to the crack and *F_Px_* is contractive along the crack due to the effect of Poisson’s ratio. *F_S_* is the induced force by the interfacial shear at the elastic-plastic boundary of the crack wake. *F_C_* is the induced force by the elastic field that originates the contact between crack flanks and the subsequent crack closure. The joint action between *F_P_* and *F_C_* induces therefore a shielding effect on the crack.

The CJP model was formulated to describe the crack fields in terms of both stresses and displacements. In the original formulation, crack tip stress fields were characterised as follows [16]:

(1) $$\begin{array}{c} \sigma_{x}=-\frac{1}{2}\left(A+4B+8F\right)r^{-\frac{1}{2}}\cos\frac{\theta }{2}-\frac{1}{2}Br^{-\frac{1}{2}}\cos\frac{5\theta }{2}-C\\ \;\;\;\;\;\;\;\;\;\;\;\;\;\;\;\;\;\;\;\;\;\;\;\;\;\;\;-\frac{1}{2}Fr^{-\frac{1}{2}}\left[\ln\left(r\right)\left(\cos\frac{5\theta }{2}+3\cos\frac{\theta }{2}\right)+\theta \left(\sin\frac{5\theta }{2}+3\sin\frac{\theta }{2}\right)\right]+O(r^{\frac{1}{2}})\\ \sigma_{y}=\frac{1}{2}\left(A-4B-8F\right)r^{-\frac{1}{2}}\cos\frac{\theta }{2}+\frac{1}{2}Br^{-\frac{1}{2}}\cos\frac{5\theta }{2}+H\\ \;\;\;\;\;\;\;\;\;\;\;\;\;\;\;\;\;\;\;\;\;\;\;\;\;\;\;+\frac{1}{2}Fr^{-\frac{1}{2}}\left[\ln\left(r\right)\left(\cos\frac{5\theta }{2}-5\cos\frac{\theta }{2}\right)+\theta \left(\sin\frac{5\theta }{2}-5\sin\frac{\theta }{2}\right)\right]+O(r^{\frac{1}{2}})\\ \sigma_{xy}=-\frac{1}{2}r^{-\frac{1}{2}}\left(A\sin\frac{\theta }{2}+B\sin\frac{5\theta }{2}\right)-Fr^{-\frac{1}{2}}\sin\theta \left[\ln\left(r\right)\left(\cos\frac{3\theta }{2}+\theta \sin\frac{3\theta }{2}\right)\right]+O(r^{\frac{1}{2}}) \end{array}$$

where *σ_x_*, *σ_y_*, and *σ_xy_* are the stress components in Cartesian coordinates, *A*, *B*, *C*, *F*, and *H* are the coefficients used to describe the stress fields around the crack tip, and *r* and *θ* are the polar coordinates assuming the crack tip as the coordinate system origin.

In addition, the CJP model was also solved in terms of displacements [16] as follows:

(2) $$2G\left(u+iv\right)=\kappa \left[-2\left(B+2F\right)z^{\frac{1}{2}}+4Fz^{\frac{1}{2}}-2Fz^{\frac{1}{2}}\ln\left(z\right)-\frac{C-H}{4}z\right]\;\;\;\;\;\;\;\;\;\;\;\;\;\;\;\;\;\;\;-z\left[-\left(B+2F\right){\bar{z}}^{-\frac{1}{2}}-F{\bar{z}}^{-\frac{1}{2}}\bar{\ln\left(z\right)}-\frac{C-H}{4}\right]\;\;\;\;\;\;\;\;\;\;\;\;\;\;\;\;\;\;\;-\left[A{\bar{z}}^{\frac{1}{2}}+D{\bar{z}}^{\frac{1}{2}}\bar{\ln\left(z\right)}-2D{\bar{z}}^{\frac{1}{2}}+\frac{C+H}{2}\bar{z}\right]$$

where *u* and *v* are the horizontal and vertical components of the displacement field, *G* = *E*/2(1 + *ν*) is the shear modulus, *E* is Young’s modulus, *ν* is Poisson’s ratio, *κ* = 3 − 4*ν* or *κ* = (3 − *ν*)/(1 + *ν*) for plane strain or plane stress conditions, respectively, and *z* is the complex coordinate. Again, the five coefficients defined in the stress fields are used to describe the displacement fields. In addition, the assumption *D* + *F* = 0 must be made in the mathematical analysis to establish a suitable asymptotic behaviour of the stress along the crack flank [16].

The CJP model defines three stress intensity factors for the characterisation of crack tip fields; an opening mode stress intensity factor *K_F_*, a retardation stress intensity factor *K_R_*, and a shear stress intensity factor *K_S_* and also provides the T-stress.

The opening stress intensity factor *K_F_* characterises the stress field that drives crack propagation and which explicitly includes the influences of plasticity-induced shielding. It is characterised by the driving force *F_A_* generated by the remote applied load, which originates crack tip stress fields traditionally characterised by classical *K_I_* but that is modified by force components derived from the stresses acting across the elastic-plastic boundary and which therefore influence the driving force for crack propagation. Therefore, unlike the classical *K_I_*, *K_F_* includes the effect of plasticity-induced crack shielding, and it is linear with the load as long as there is no shielding effect. *K_F_* is defined from the asymptotic limit of *σ_y_* as *x* → +0, along *y* = 0, i.e., towards the crack tip on the crack plane ahead of the crack tip:

(3) $$K_{F}=\underset{r\to 0}{\lim}\left[\sqrt{2\pi r}\left(\sigma_{y}+2Fr^{-1/2}\ln r\right)\right]=\sqrt{\frac{\pi }{2}(A-3B-8F)}$$

The retardation effect intensity factor *K_R_* is characterised by forces applied in the plane of the crack and which provide a retarding effect on fatigue crack growth. *K_R_* is defined from *σ_x_* in the limit as *x* → −0, along *y* = 0, i.e., towards the crack tip along the crack flank:

(4) $$K_{R}=\underset{r\to 0}{\lim}\left[\sqrt{2\pi r}\sigma_{x}\right]=-{\left(2\pi \right)}^{3/2}F$$

The shear stress intensity factor *K_S_* is characterised by compatibility-induced shear stress along the plane of the crack at the interface between the plastic enclave and the surrounding elastic stress field and is derived from the asymptotic limit as *x* → −0, along *y* = 0, i.e., towards the crack tip along the crack wake:

(5) $$K_{S}=\underset{r\to 0}{\lim}\left[\sqrt{2\pi r}\sigma_{xy}\right]=\pm \sqrt{\frac{\pi }{2}(A+B)}$$

A positive sign indicates *y* > 0, and a negative sign that *y* < 0.

The T-stress, which is found as components *T_x_* in the *x*-direction and *T_y_* in the *y*-direction is given by:

(6) $$T_{x}=-C\;y=-H$$

This model of crack tip fields is somewhat controversial, so the present paper is primarily oriented towards demonstrating the capability of the CJP model to investigate the effects of plasticity on growing fatigue cracks through the quantitative evaluation of plasticity-induced crack shielding and the retardation effect induced on the crack growth rates due to the application of single overloads as well as the estimate of the size and shape of the plastic zone at the crack tip.

In addition, the difference between *K_F_* and *K_R_* at maximum and minimum load explicitly provides the effective crack driving force, Δ*K_eff_* = (*K_F_* − *K_R_*)*_max_* − (*K_F_* − *K_R_*)*_min_*. According to this, the crack shielding effect will be investigated from the calculation and analysis of these two stress intensity factors, and the mathematical formulation describing the crack tip fields defined by the five CJP coefficients will be used to provide predictions for the extension of the plastic zone generated at the tip of propagating fatigue cracks. The experimental results obtained from the stress intensity factor analysis are compared with nominal *K_I_* values obtained from the following standard expressions for the aluminium (Equation (7) in [26]) and titanium (Equation (8) in [27]) specimens:

(7) $$K_{t}=\frac{P}{t\cdot W}\sqrt{a}\frac{2\left(2+\frac{a}{W}\right)}{{\left(1-\frac{a}{W}\right)}^{3/2}}\frac{1}{\sqrt{\frac{a}{W}}}F_{2}\left(\frac{a}{W},\frac{h}{W},\frac{d}{h}\right)$$

(8) $$K_{I}=\frac{P}{t\sqrt{W}}\frac{2+\frac{a}{W}}{{\left(1-\frac{a}{W}\right)}^{3/2}}\left(0.886+4.64\frac{a}{W}-13.32{\left(\frac{a}{W}\right)}^{2}+14.72{\left(\frac{a}{W}\right)}^{3}--5.6{\left(\frac{a}{W}\right)}^{4}\right)$$

where *P* is the applied load, *a* is the crack length, *t* and *W* are the thickness and width of the specimen, respectively, and *F_2_* is a non-dimensional function whose value is estimated graphically from Tada et al. [26].

## 3. Materials and Experimental Details

**Figure 2:**
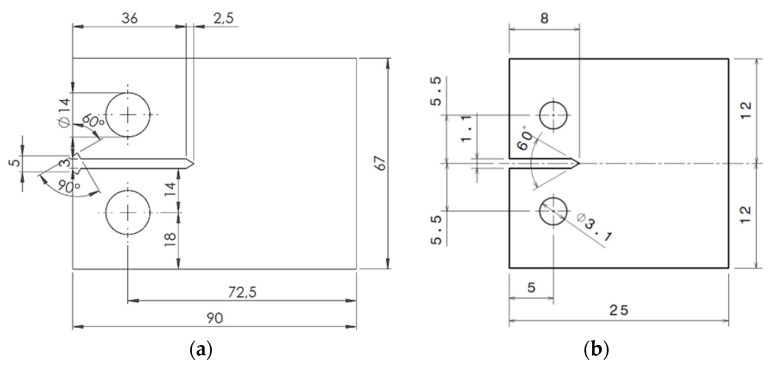
Dimensions (mm) of the CT specimens tested: (**a**) 2024-T3 aluminium alloy, (**b**) commercially pure titanium.

Six CT specimens (geometry and dimensions as shown in Figure 2) were manufactured from sheets of 2024-T3 aluminium alloy and commercially pure titanium with a thickness of 2 mm and 1 mm, respectively. Aluminium and titanium alloys are some of the most common materials used in industrial applications, airframes among others. Furthermore, 2024-T3 aluminium and commercially pure titanium are particularly relevant to the aerospace industry due to their high strength and good resistance to fatigue crack propagation and corrosion. In addition, two different dimensions were selected because they were used for different tasks. Thus, a higher geometry was used in the case of the aluminium specimens in order to have a higher range of crack lengths since they were tested to study crack shielding and the effect of overloads during fatigue crack propagation. In the case of the titanium specimens, they were tested with a higher magnification to quantify the plastic zone at the crack tip and, for this reason; a smaller geometry was more convenient to investigate this phenomenon.

Table 1 presents the more relevant mechanical properties of the two materials tested in this work.

The aluminium specimens were used to explore crack shielding, while the titanium specimens were used to estimate the crack tip plastic zone. The experimental conditions for the fatigue tests are detailed in Table 2.

Two aluminium specimens (AACT1 and AACT2) were tested at two different *R*-ratio levels to study plasticity-induced crack shielding under constant amplitude loading and the other two specimens (AACT3 and AACT4) were subjected to two different overload levels to investigate the effects induced on fatigue crack propagation. Thus, stress ratio values of 0 and 0.5 were selected to be representative in order to explore their influence in the study of the crack shielding effects induced by plasticity. For the study of the overload effects, a stress ratio of 0 was selected to explore at the same time the retardation effect induced on fatigue crack growth due to the natural propagation of the crack at a low *R*-ratio and also due to the application of different overloads. In the case of the titanium specimens, both of them were tested for the quantitative evaluation of the plastic zone on growing fatigue cracks at constant amplitude (TiCT1) and at variable amplitude due to the application of an overload (TiCT2). A high stress ratio value was selected to avoid any possible shielding effect induced during crack propagation at a low ratio. In this case, 0.6 was the stress ratio value selected, but 0.5 could have been chosen. Therefore, the purpose of this work is to combine the DIC technique together with the CJP model to experimentally evaluate the effects induced by the plastic enclave generated around a fatigue crack in two different ways: those induced by the natural propagation of the crack and those induced by the application of overloads.

It is known that the specimen thickness influences the size and shape of the plastic zone [10]. Thinner specimens are related to a plane stress state whereas thicker specimens are related to a plane strain state. In addition, DIC is an optical technique where the conditions at the specimen surface are analysed. According to this, thin specimens were tested in this work to ensure plane stress conditions on the specimens. Nevertheless, thick specimens can be also tested with DIC but only the conditions at the specimen surface would be analysed. In this case, the conditions in the surface of the specimens must be experimentally studied with a different technique (e.g., X-ray diffraction [28]) or numerically by a finite element analysis.

**Figure 3:**
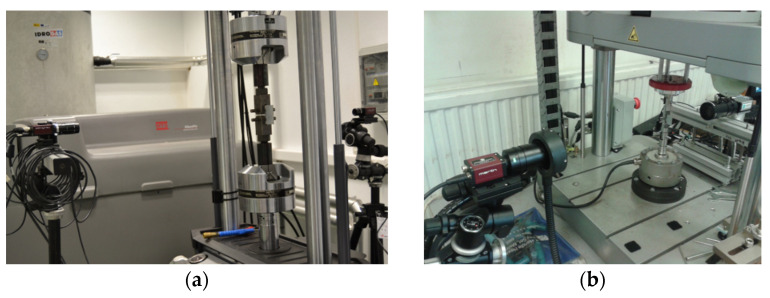
Experimental setup used for fatigue testing and data acquisition: (**a**) 2024-T3 aluminium alloy, (**b**) commercially pure titanium.

Similar experimental setups were used for fatigue testing and data acquisition in both materials (Figure 3).

Fatigue tests at 10 Hz were conducted on the aluminium specimens using a 100 kN servohydraulic machine (MTS, model 370.10, Madrid, Spain), while a 3 kN electrodynamic machine (Instron, model ElectroPuls E3000, High Wycombe, UK) was used for the titanium specimens applying the same frequency. Regarding the experimental observation, two cameras were used, one placed perpendicularly at each side of the specimens (as shown in Figure 3). One camera was used to acquire the sequence of images to measure the displacement fields with DIC and an additional camera was used to assist in tracking the crack tip. For implementing DIC, a charge-coupled device (CCD) camera (Allied Vision Technologies, model Stingray F-504B/C, Stadtroda, Germany) with a 75 mm lens was used in the case of the aluminium specimens (resolution of 33 μm/pixel), while a CCD camera (Allied Vision Technologies, model Marlin F-146B/C, Stadtroda, Germany) fitted with a macro-zoom lens (Edmund Optics, model MLH-10X) was used for the titanium specimens (resolution of 13.5 μm/pixel). In addition, the specimen surface used for this task was treated by spraying a black speckle with an airbrush over a white background.

**Figure 4:**
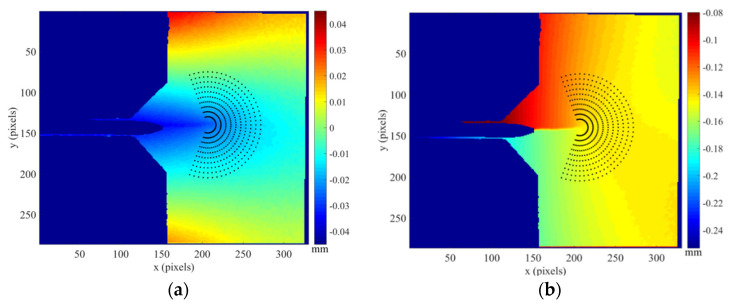
Annular collection of data points around the crack tip overlapped on the displacement fields for a 35 mm crack and a load level of 600 N corresponding to the aluminium specimen subjected to a 125% of overload (AACT4): (**a**) Horizontal displacements, (**b**) vertical displacements.

During fatigue testing, a sequence of images was acquired at different load levels along a full loading cycle by periodically pausing the fatigue cycling and recording speckled images at each load level. A cross-correlation algorithm [4] implemented in the Vic-2D system [29] developed by Correlated Solution^®^ company (Columbia, SC, USA) was then used to process the acquired sequences of images. The images were processed using 21 and 5 pixels, respectively, for the facet size and the overlap. Typical maps obtained by DIC of the horizontal and vertical displacement fields for a 35 mm crack and 600 N are shown in Figure 4.

## 4. Experimental Methods

As mentioned above in this work, the CJP model is combined with DIC to investigate the effects of plasticity on growing fatigue cracks through the evaluation of plasticity-induced crack shielding and the retardation effect induced on fatigue crack propagation due to the application of overloads. In addition, the ability of the CJP model to predict both the shape and size of the plastic zone at the tip of growing fatigue cracks is also studied by comparing these predictions with the experimental estimates obtained by applying the von Mises yield criterion. According to this, in this section, the two methodologies used to explore both phenomena, the crack shielding effect and the estimate of the plastic zone, are described.

### 4.1. Calculation of Stress Intensity Factors

The shielding effects induced by plasticity generated around a growing fatigue crack are evaluated from the calculation of the stress intensity factors by analysing the displacement fields measured by DIC. The multi-point over-deterministic method developed by Sanford and Dally [30] formed the basis for the calculation of the stress intensity factors. The CJP model is valid only for the elastic field dominated by the singularity around the crack tip, and hence, it was necessary to identify the near-tip zone where experimental data satisfied this validity requirement. An annular mesh was therefore defined, as shown in Figure 4, on the basis of inner and outer radii. The extension of the inner radius was defined to avoid including plastic deformation at the crack tip, while the outer radius was defined to be within the singularity-dominated region, which was identified by observing the vertical displacement map where the fringes orientation of the map becomes straight and perpendicular to the crack, from which the displacement orientation changes due to the specimen edge effects. The region of data collection does not encompass the crack flanks to avoid collecting data where the elastic crack tip stress field no longer describes the stress state. In addition, a relevant aspect of the calculation process is the accurate location of the crack tip. According to this, the mean and variance of a statistical assessment of the quality of the fit between the analytical solution of the crack tip fields and the experimental data were used for the optimisation of the crack tip position. Thus, that point in the image with the lowest values of the mean and the variance gives the correct location of the crack tip.

Then, Equation (2) is used to fit the processed image data, where the displacement fields and the five CJP coefficients are directly related, solving a linear system of equations to obtain the unknown coefficients. These five coefficients are then used to determine the stress intensity factors and the T-stress defined in the CJP model (Equations (3)–(6)). In addition, an error function (see Equation (9)) was defined to optimise the fit between the experimental data and the mathematical expressions describing the crack tip displacement fields. This error function was minimised using a value of 10^−5^.

(9) $$\left\{\begin{array}{c} g_{u,j}\\ g_{v,j} \end{array}\right\}=\left\{\begin{array}{c} f_{u,j}(A,B,C,F,H,z,G,\nu )\\ f_{v,j}(A,B,C,F,H,z,G,\nu ) \end{array}\right\}-\left\{\begin{array}{c} u_{j}\\ v_{j} \end{array}\right\}=0$$

The subscript *j* indicates the value of the error function evaluated at the *j*th data point with polar coordinates (*r*, *θ*).

### 4.2. Evaluation of Crack Tip Plastic Zone

Besides the evaluation of plasticity-induced crack shielding during fatigue crack growth by using the CJP model, the ability of this model to predict the size and shape of the crack tip plastic zone is also investigated. In this section, the two methods used to evaluate the plastic zone size and shape are described. Thus, in the first method the plastic zone is estimated by applying the von Mises yield criterion from the differentiation of the displacement fields obtained by experiment, while in the second method, the plastic zone is predicted by using the mathematical formulation of the CJP model. A comparison is therefore performed between both estimates of the plastic zone.

#### 4.2.1. Experimental Estimate of Crack Tip Plastic Zone

The application of the von Mises yield criterion forms the basis of this method and consists of differentiating the experimental displacement maps for later identifying the plastic stress field by using the yield stress of the material. The experimental displacement fields analysed in this work have been obtained by applying 2D DIC. The implementation of this method is described in the following paragraph.

The experimental measurement of the horizontal and vertical displacement fields is the first step of the methodology. In the second step, the strain fields at the crack tip are determined by differentiation of the displacement fields using the Green–Lagrange strain tensor [31] since it considers second-order terms and is hence more accurate than expressions that use only first-order terms. This strain tensor is given as follows:

(10) $$\left(\begin{array}{c} \varepsilon_{xx}\\ \varepsilon_{yy}\\ \varepsilon_{xy} \end{array}\right)=\left(\begin{array}{c} \frac{\partial u}{\partial x}\\ \frac{\partial v}{\partial y}\\ \frac{\partial u}{\partial y}+\frac{\partial v}{\partial x} \end{array}\right)+\frac{1}{2}\left(\begin{array}{c} \frac{\partial u}{\partial x}\\ 0\\ \frac{\partial u}{\partial y} \end{array}\begin{array}{c} 0\\ \frac{\partial u}{\partial y}\\ \frac{\partial u}{\partial x} \end{array}\begin{array}{c} \frac{\partial v}{\partial y}\\ 0\\ \frac{\partial v}{\partial y} \end{array}\begin{array}{c} 0\\ \frac{\partial v}{\partial y}\\ \frac{\partial v}{\partial x} \end{array}\right)\left(\begin{array}{c} \frac{\partial u}{\partial x}\\ \frac{\partial u}{\partial y}\\ \frac{\partial v}{\partial x}\\ \frac{\partial v}{\partial y} \end{array}\right)$$

Therefore, the Green–Lagrange tensor includes the linear infinitesimal tensor for small strains (first order term in Equation (10)) and the nonlinear terms for large strains (second order term in Equation (10)).

Once the strains have been obtained, the stress fields can be calculated using Hooke’s law:

(11) $$\sigma_{xx}=\frac{E}{1-\nu^{2}}\left(\varepsilon_{xx}+\nu \varepsilon_{yy}\right)\;\sigma_{yy}=\frac{E}{1-\nu^{2}}\left(\varepsilon_{yy}+\nu \varepsilon_{xx}\right)\;\sigma_{xy}=2G\varepsilon_{xy}$$

**Figure 5:**
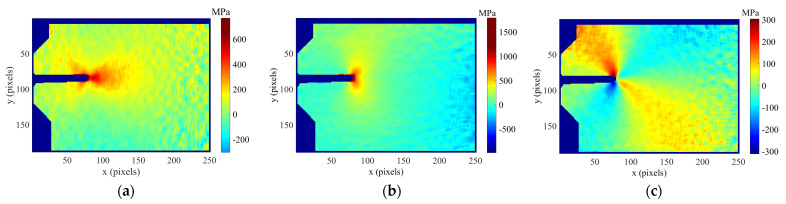
Stress maps for TiCT2 specimen obtained from the strain fields at an 8.3 mm crack and a load of 750 N: (**a**) *σ_xx_*, (**b**) *σ_yy_*, (**c**) *σ_xy_*.

Figure 5 shows the stress maps (*σ_xx_*, *σ_yy_, σ_xy_*) calculated from the strain fields for the titanium specimen subjected to a 50% overload (TiCT2) for a crack length of 8.3 mm and a load level of 750 N.

**Figure 6:**
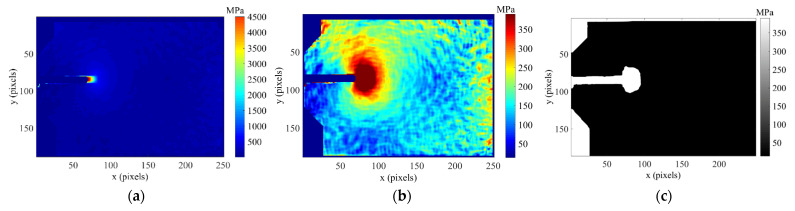
Von Mises equivalent stress map for TiCT2 specimen for an 8.3 mm crack and a load level of 750 N: (**a**) Directly obtained by implementing Hooke’s law, (**b**) Stress map using the yield stress as the threshold value, (**c**) Processed to show the extension of the plastic zone around the crack tip as the white region.

The equivalent stress is then calculated by applying a suitable yield criterion. In this work, the von Mises yield criterion was used as it has been shown to be the most appropriate for a ductile material [32,33]. Figure 6a is the von Mises equivalent stress map obtained from the stress fields shown in Figure 5 for specimen TiCT2 at a crack length of 8.3 mm and a load level of 750 N.

The size and shape of the plastic zone are estimated by identifying the region where the yield criterion is met, i.e., where the equivalent stress is equal to the yield stress of the material. Thus, the equivalent stress map using the yield stress as the threshold value is shown in Figure 6b, while in Figure 6c the equivalent stress has been converted to a binary black and white format using a greater than or equal operator (>=) to show the extension of the plastic zone around the crack tip.

In this work, the area of the plastic zone is considered a variable that contains useful information on crack growth, and the method clearly provides an efficient way for making quantitative measurements of the monotonic plastic zone. Once the plastic zone extension has been identified, its area was calculated using the function “bwarea”, implemented in Matlab^®^ (version R2017a). This function is used to estimate the area of objects in a black-white binary image.

#### 4.2.2. Predictions of Crack Tip Plastic Zone

As mentioned above, the ability of the CJP model to predict both the size and shape of the plastic zone generated at the tip of propagating fatigue cracks is also investigated in this paper. Therefore, the predicted plastic zones by the CJP model are compared with the experimental plastic zones obtained by implementing the method described above.

In the literature, the two most common approaches to estimate the size of the crack tip plastic zone are the Irwin and Dugdale estimates [10]. Both methods lead to simple estimates of the size of the plastic zone since are based on linear elastic fracture mechanics (LEFM). However, the shape of the plastic zone predicted by these approaches does not agree with that experimentally observed in metals [10]. The combination of applying a yield criterion together with an analytical model for the characterisation of crack tip fields to estimate both the extension and shape of the plastic zone as a function of the angle around the crack tip can constitute a more useful procedure. The CJP model is used in this work to find the plastic zone extension around the crack tip since as described in Section 2, crack tip fields are characterised by a set of coefficients (*A*, *B*, *C*, *F*, *H*) and the polar coordinates (*r*, *θ*) of data points around the crack tip. According to this, the CJP model seems very appropriate to estimate the plastic zone size and shape as a function of the angle around the crack tip. Determining the five coefficients defined by the CJP model constitutes, therefore, the first step for estimating the plastic zone extension from the analysis of the crack tip displacement fields measured with DIC using the method described in Section 4.1 based on collecting a set of data points around the crack tip to calculate the stress intensity factors.

Crack tip stress fields are then evaluated from the CJP coefficients previously determined. The von Mises equivalent stress map is therefore obtained as a function of the CJP coefficients and the polar coordinates around the crack tip since the stress fields are described by these parameters as described above. An error function is then defined to represent the difference between the equivalent von Mises stress and the uniaxial yield stress of the material:

(12) $$f_{error}=\sigma_{eq}\left(A,B,C,F,H,r,\theta \right)-\sigma_{ys}=0$$

**Figure 7:**
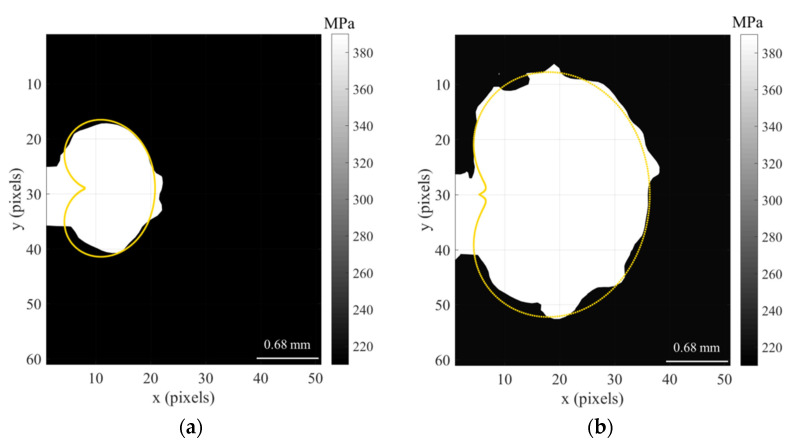
Experiment results from DIC and predicted by the CJP model plastic zone shapes obtained applying the von Mises yield criterion for two different crack lengths (6.1 (**a**) and 9.2 mm (**b**)), corresponding to the titanium specimen tested at constant amplitude loading cycle (TiCT1).

Solving this function for all angles around the crack tip defines the yield boundary contour, which can then be used to determine the required information on both the size and shape of the plastic zone around the crack tip. Figure 7 shows a comparison between the experimental estimate of the crack tip plastic zone (white area) and the prediction by the CJP model (orange line) for the titanium specimen tested at constant amplitude loading cycle (TiCT1) at two different crack lengths, 6.1 mm (Figure 7a) and 9.2 mm (Figure 7b).

There is clearly a high level of agreement between both results from a qualitative point of view. Regarding a quantitative comparison, it is carried out through the plastic zone area as mentioned above, which is shown in the following section.

## 5. Results and Discussion

In this section, the experimental results obtained both on the evaluation of the effects of plasticity on growing fatigue cracks and on the estimate of the size and shape of the crack tip plastic zone are analysed and discussed. Thus, both phenomena are analysed in the case of propagating cracks at constant amplitude fatigue and variable amplitude due to the application of overloads.

### 5.1. Results on Plasticity-Induced Shielding Effects

**Figure 8:**
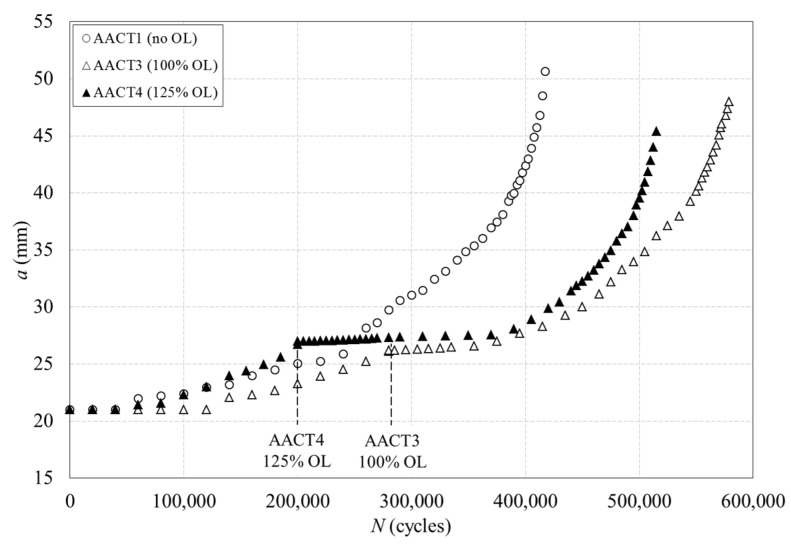
Evolution of the crack length with the number of cycles for the aluminium specimens tested at *R* = 0: AACT1 specimen at constant amplitude fatigue and AACT3 and AACT4 specimens subjected to a 100% and 125% of overload level, respectively.

Four 2024-T3 aluminium alloy specimens (see Table 2) were tested to investigate the effects of plasticity induced during fatigue crack propagation at constant amplitude and under overloads. Figure 8 shows the evolution of the crack length with the number of cycles for the three aluminium specimens tested at *R* = 0: AACT1 specimen at constant amplitude fatigue and AACT3 and AACT4 specimens subjected to 100% and 125% overload levels, respectively, for similar crack lengths (26.1 mm for AACT3 and 26.7 mm for AACT4). A gradual trend in the crack propagation is observed for the specimen tested at constant amplitude fatigue. However, a retardation effect on the crack propagation was induced by the two applied overloads, this retardation effect being longer in the case of the highest overload. Thus, 135,000 cycles was the influence of the retardation effect induced by 100% overload (1.2 kN), which corresponded to 2.1 mm for the crack length increment; while 220,000 cycles was the retardation influence induced by 125% overload (1.35 kN), corresponding to an increment of 3.4 mm for the crack length. This deceleration of fatigue crack growth was therefore a direct consequence of the plastic deformation increase at the crack tip originated by the overloads.

**Figure 9:**
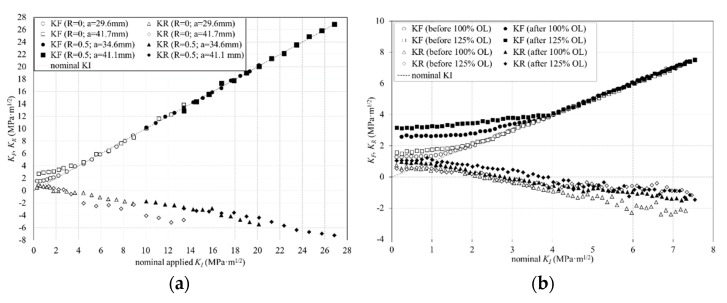
*K_F_* and *K_R_* values through a loading half-cycle for the aluminium specimens: (**a**) For two fatigue cracks in the specimens tested at constant amplitude fatigue; (**b**) immediately before (black symbols) and after (white symbols) the applied overloads in the case of the specimens subjected to overloads of 100% and 125%.

The interest in this work lies in investigating the ability of the CJP model for the accurate characterisation of fatigue crack growth and assisting in shedding some light on the causes of the observed changes in the crack growth rates due to the application of an overload, i.e., whether or not these changes can be explained solely by the shielding effects induced by the plastic enclave generated around a fatigue crack. According to this, the variation of *K_F_* and *K_R_* values through a loading half-cycle and along the crack length is analysed and discussed since the effective crack driving force is estimated as the difference between these two stress intensity factors. Figure 9a shows *K_F_* and *K_R_* values during a loading half-cycle as a function of the nominal *K_I_* values at two different crack lengths for the two specimens tested at constant amplitude fatigue (AACT1 at *R* = 0 and AACT2 at *R* = 0.5).

In the case of the specimen tested at a high *R* ratio (black symbols), *K_F_* values closely follow the trend in the nominal *K_I_* values. In contrast, in the case of the specimen tested at a low stress ratio, *K_F_* deviates above the nominal *K_I_* once the values fall below approximately 2.2 MPa·m^1/2^ and 4.5 MPa·m^1/2^ for crack lengths of 29.6 mm and 41.7 mm, respectively. This behaviour is similar to that reported by Elber [1] when he defined plasticity-induced crack closure. Elber noticed an anomaly in the elastic compliance of fatigue specimens, where the compliance agreed with that obtained from standard expressions for fracture mechanics specimens at high loads, whilst at low loads, the compliance was close to that for an uncracked specimen. He attributed this change in compliance to contact between crack flanks (i.e., crack closure) in the lower part of the load cycle as a consequence of the residual tensile plastic deformation left in the wake of a fatigue crack. The conception of the CJP model postulates that wake contact is not the only mechanism that leads to a shielding effect on the crack, contributing to the compatibility-induced stresses at the elastic-plastic boundary as a further component. According to this, the stress-intensity factor value at which the crack opens (*K_op_*) can be inferred from the *K_F_* trend as that value corresponds to the minimum values of the loading range. Therefore, attending to the *K_F_* values indicated above, a higher shielding effect is induced as the crack grows. It is clear then that no shielding effect was induced on the crack in the case of the specimen tested at a high *R*-ratio, with the non-existence of a *K_op_* value.

In the case of the specimen tested at *R* = 0.5, from the analysis of *K_R_*, all values are negative, following a gradual increase (in negative terms) through the complete loading cycle. However, for the specimen tested at low stress ratio, a change in the *K_R_* sign is observed approximately for the same portion of the cycle where a change in the *K_F_* trend was observed. This change in trends of *K_F_* and *K_R_* reveals hence the existence of shielding effects on the crack at low load levels, dividing the loading cycle into two intervals, that at which the crack is closed and that at which it is open.

Besides the study of the crack shielding effects during fatigue crack growth at constant amplitude, the effects of different applied overloads on fatigue-propagating cracks are also studied. Figure 9b shows *K_F_* and *K_R_* values along a loading half-cycle occurring immediately prior to (blank symbols) and immediately after (white symbols) the overload cycle for the two specimens subjected to a single overload (100% overload for AACT3 specimen and 125% overload for AACT4 specimen). It is clear in this figure that the applied overloads produced a substantial increase in the magnitude of *K_F_* and *K_R_* values in the regions where a trend change or a sign change is observed for *K_F_* and *K_R_*, respectively. This change in *K_F_* and *K_R_* values occurs at a higher proportion of the loading half-cycle showing that the shielding effect induced on the crack has increased and *ΔK_eff_* value has decreased after the overload, with the subsequent fatigue life increase of the analysed component.

**Figure 10:**
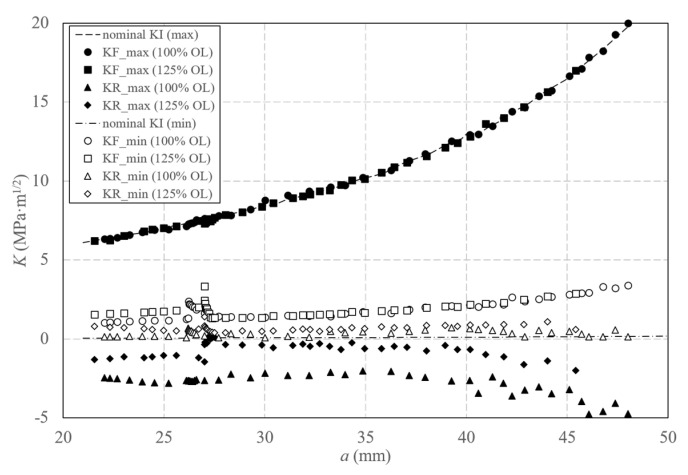
Variation in *K_F_* and *K_R_* values with the crack length corresponding to the minimum (white symbols) and maximum (black symbols) applied loads for the aluminium specimens subjected to 100% and 125% of overloads.

Moreover, the evolution of *K_F_* and *K_R_* with crack length at maximum (black symbols) and minimum (white symbols) loads in the case of the two applied overloads is shown in Figure 10.

For the conditions at maximum load, *K_F_* values agree with nominal *K_I_* values, not observing any change in its trend; while *K_R_* values are negative. However, the most interesting information is given by the conditions at minimum load. All *K_F_* values are higher than nominal *K_I_* values, highlighting plasticity-induced crack shielding effects for all crack lengths considering the above analysis corresponding to the *K_F_* plots along a loading half-cycle. In addition, this crack shielding effect is increased immediately after the application of the overloads, illustrated by a punctual increase in *K_F_* values and a subsequent gradual decrease as cracks grow until reaching the growth rate before applying the overloads. Thus, a bigger shielding effect is observed in the case of the specimen subjected to a higher overload, with a longer retardation effect on fatigue crack growth induced by the increase in plastic deformation arising from the overload. A similar behaviour to *K_F_* is observed for *K_R_*, being in this case less relevant since dealing with smaller values.

**Figure 11:**
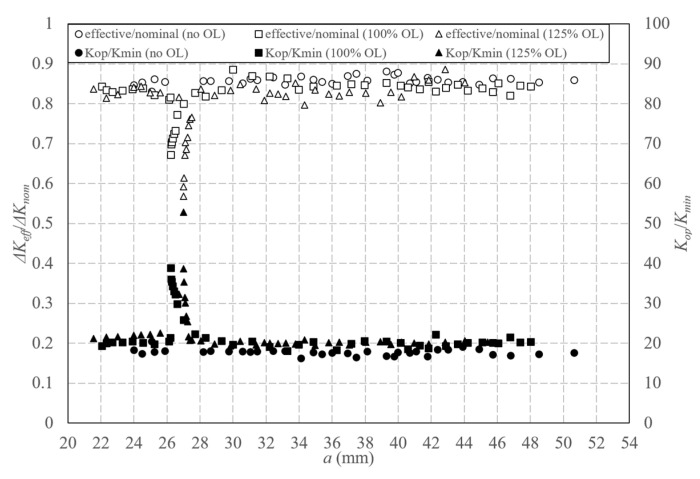
Variation in the ratios Δ*K_eff_/*Δ*K_nom_* and *K_op_/K_min_* with the crack length for the three aluminium specimens. The ratio *K_op_/K_min_* has been represented in a secondary vertical axis.

The shielding effect induced on a growing fatigue crack due to an applied overload can be more usefully illustrated by plotting the ratio between the effective and nominal ranges of the stress intensity factor (white symbols) and the ratio between the opening and minimum stress intensity factor (black symbols) along the crack length, as has been jointly plotted in Figure 11.

Those results corresponding to the test at constant amplitude loading have been also plotted to establish a comparison with the results in the case of overloads. From the analysis of Δ*K_eff_*/Δ*K_nom_*, several trends are observed in this figure. Firstly, most of the values are around 0.85, highlighting a plasticity-induced shielding effect since the three specimens were tested at a stress ratio of 0. This conclusion had been established above from the analysis of *K_F_*, now supported by these results on the effective crack growth rates. In addition, most *K_op_*/*K_min_* values are around 20; showing that a load higher than the minimum one must be applied to open the fatigue crack. This result illustrates again the shielding effect induced by the plastic enclave generated around a growing fatigue crack. Other trends are observed from the results obtained by the applied overloads; regarding Δ*K_eff_*/Δ*K_nom_*, a punctual decrease is observed in their values immediately after the application of the overloads, being 0.67 and 0.57, respectively, for the specimens subjected to 100% and 125% overloads. After this substantial decrease, there is a gradual increase in Δ*K_eff_*/Δ*K_nom_* values as cracks grow until reaching the trend before the application of overloads. This behaviour reveals an increase in plasticity-induced crack shielding during the overload influence, the shielding effect being lower in the case of the specimen with a lower overload. Therefore, a clear retardation effect was induced during fatigue crack propagation, being higher in the case of the biggest overload. In contrast, there is a punctual increase in *K_op_*/*K_min_* values immediately after the overloads, followed by a gradual decrease as cracks grow until reaching again the trend before the overloads. In this case, this increase supposed values of 39 and 53 for the 100% and 125% overloads, respectively. An inverse behaviour is therefore observed for both ratios, being a reduction in the case of the effective crack growth rates and an increase for the load level to be applied to open the crack. These two behaviours have in common plasticity-induced shielding effects arising for two reasons: first, due to fatigue tests at a low stress ratio (*R* = 0 in this case), and second, due to the application of overloads.

### 5.2. Results on Crack Tip Plastic Zone Estimate

**Figure 12:**
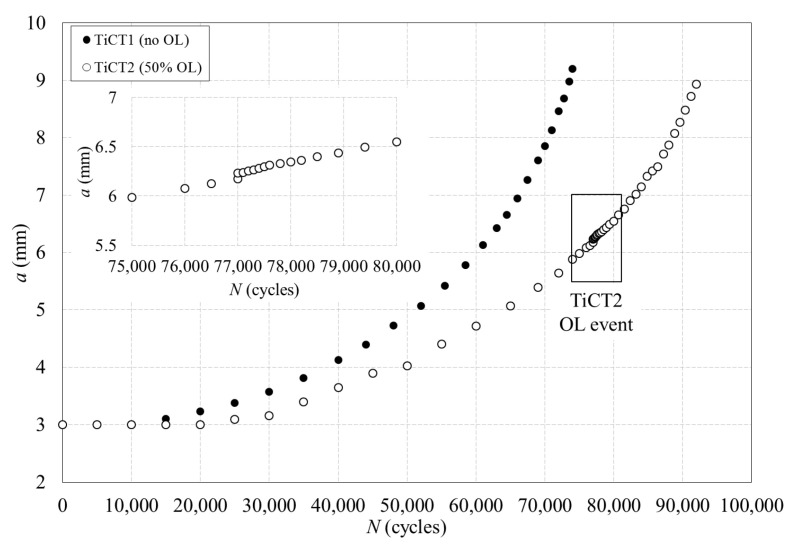
Evolution of crack length with the number of cycles for the titanium specimens tested at *R* = 0.6: TiCT1 specimen tested at constant amplitude cycle and TiCT2 specimen subjected to a 50% of overload level. The overload event has been enlarged in the specimen TiCT2.

Besides the evaluation of the shielding effects induced by the plastic enclave generated around growing fatigue cracks, the ability of the CJP model to predict and quantify the size and shape of the plastic zone at the tip of a fatigue crack is also investigated in the present work. The study has been performed on two CT commercially pure titanium specimens (Figure 2b) tested both at constant amplitude fatigue and under the application of an overload (see conditions in Table 2). The evolution of crack length with the number of cycles is shown in Figure 12, where the overload event has been enlarged to assist in its observation.

**Figure 13:**
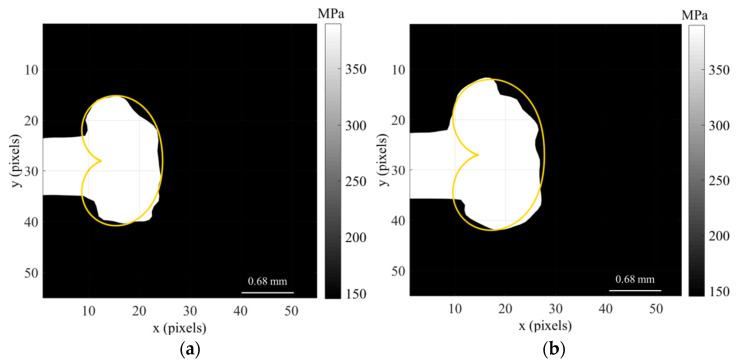
Comparison between the experimental and predicted plastic zone shapes corresponding to the cycles immediately before (**a**) and after (**b**) the application of the overload for the titanium specimen subjected to a 50% overload level (TiCT2).

A light change in the crack growth rate was induced in the case of the specimen subjected to a 50% overload, where its influence extended over 1900 cycles (corresponding to a crack length increment of 0.27 mm). Nevertheless, the interest here is to explore if the CJP model is able to characterise both the size and shape of the plastic zone. Thus, the CJP model predictions are compared with the experimental plastic zone estimated from the differentiation of the displacement fields measured with DIC as indicated in Section 4.2. Figure 13 compares the plastic zone size and shape obtained immediately prior to the overload and immediately after the overload cycle.

In the first observation, the great level of agreement obtained for the experimental (white region) and the predicted by the CJP model plastic zone (yellow contour) is clear. The overload was applied at a crack length of 6.2 mm, producing an increase in the area of the crack tip plastic zone after the applied load returned to the pre-overload level. The results obtained for the area of the plastic zone before the application of the overload were the following: 1.49 mm^2^ for the experimental estimate and 1.55 mm^2^ for the CJP model prediction. This supposes a difference in the plastic zone area of 4% for the pre-overload conditions. In the case of post-overload conditions, the results were 2.14 mm^2^ for the experimental area and 2.29 mm^2^ for the predicted area, corresponding to an increase of 43.6% and 47.7%, respectively, regarding the pre-overload conditions. All these results show the great potential of the CJP model to characterise the crack tip plastic zone both qualitatively from the direct observation of its shape and quantitatively through the area calculated as a parameter to consider its size.

**Figure 14:**
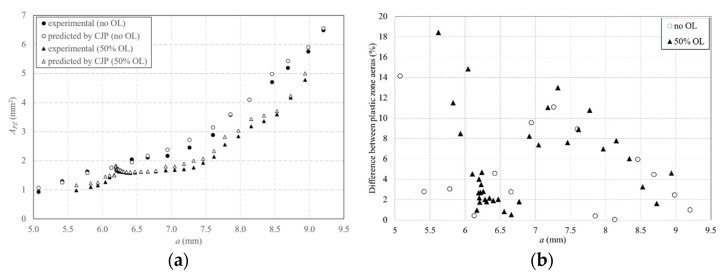
Comparison between the experimental and CJP predictions of plastic zone area as a function of the crack length for the titanium specimens: (**a**) Values of the plastic zone area and (**b**) difference, as a percentage, between both plastic zone areas.

Moreover, the observed change in the plastic zone size due to the applied overload can be tracked as the crack grows during the event. Such data are shown and compared in Figure 14a, where the experimental and predicted data for the plastic zone area (*A_PZ_*) with the crack length for both CT specimens have been plotted.

**Figure 15:**
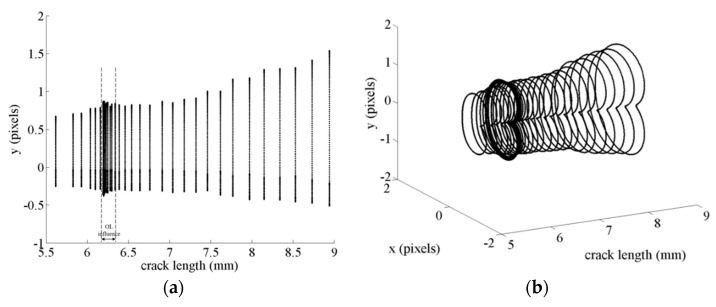
Illustrations of the evolution along crack length of the plastic zone shapes at the crack tip predicted by the CJP model for the titanium specimen subjected to a 50% overload: (**a**) 2D view and (**b**) 3D view.

In all cases, there is a very good level of agreement between the experimental data and the predictions obtained with the CJP model. Prior to the overload, the plastic zone area increases steadily along the crack length, with a significant increase with the application of the overload. During continued post-overload cycles, a gradual decrease in the plastic zone area is observed towards the pre-overload trend in the data. Thus, the same overload influence region in crack length as that above indicated when *a* vs. *N* graph was analysed is observed in this figure. In addition, the difference between the experimental and predicted plastic zone area for both specimens is shown in Figure 14b. Most of the data are below 10%, observing the highest differences for shorter cracks, where a higher error can be made due to a lower size in the plastic zone. The evolution of the plastic zone as a function of crack growth during the overload event can also be easily visualised by plotting sequential CJP model predictions. Figure 15 presents two illustrations of this shape evolution, giving a 2D and 3D representation. Figure 15a presents a line illustration showing the maximum size in the vertical *y* direction, marking the overload influence region; while Figure 15b provides the clearest illustration of plastic zone development through a 3D view.

## 6. Conclusions

The work reported in the present paper has clearly demonstrated the great potential of the CJP model of crack tip fields to experimentally evaluate the plasticity-induced shielding effects on growing fatigue cracks and to characterise the size and shape of the plastic zone generated at the crack tip during fatigue crack propagation. These phenomena have been studied at different crack growth conditions, under amplitude constant fatigue and under overload conditions. The model has been successfully applied by analysing displacement fields experimentally measured by DIC on propagating fatigue cracks in different CT specimens made of 2024-T3 aluminium alloy and commercially pure titanium.

The shielding effects induced by plasticity have been evaluated on 2024-T3 aluminium alloy specimens from the analysis of *K_F_* and *K_R_* stress intensity factors defined by the CJP model. The variation of the effective range of stress intensity factor (Δ*K_eff_*) and the opening value of stress intensity factor (*K_op_*) with crack length have clearly shown retardation effects on propagation rates induced by the plastic enclave generated around the cracks. These plasticity-induced crack shielding effects and the consequent retardation effect on fatigue crack growth have been quantified in two different ways: that induced by the natural propagation of the crack and that induced by the application of overloads. A steady shielding effect was induced on growing fatigue cracks at *R* = 0 due to a gradual increase in plasticity as cracks grow. In addition, the applied overloads induced a higher shielding effect due to the plasticity increase, the retardation effect being longer in the case of the highest overload. The results on the retardation effects on propagating fatigue cracks arising from plasticity-induced shielding demonstrate that the mathematical formulation of the CJP model considering plasticity influences on a fatigue crack accurately characterises crack tip fields.

The work on the crack tip plastic zone has been performed on commercially pure titanium specimens. Thus, the experimental estimates of the plastic zone have been compared with predictions obtained from the CJP model at constant amplitude fatigue and due to the application of an overload. The experimental estimates were obtained from the combination of a yield criterion with the differentiation of the displacement fields quantified by DIC to find the shape and size of the plastic zone during fatigue crack propagation. A very good level of agreement was obtained between the predictions of the CJP model and the experimental results, highlighting the great potential of the CJP model for the accurate characterisation of the size and shape of the plastic zone generated at the tip of propagating fatigue cracks through its formulation that considers the influences of the plastic enclave around a fatigue crack on the elastic stress fields ahead of the crack tip.

Results presented in this work are intended to contribute to a better comprehension of the shielding effects of the plastic enclave generated around a fatigue crack. The CJP model has been demonstrated to be a valuable tool in fracture mechanics when plasticity plays a significant role, such as plasticity-induced crack shielding and crack tip plasticity.

In summary, the following concluding remarks have been achieved in this work:

- The CJP model shows great potential to quantitatively evaluate the shielding effects induced on growing fatigue cracks at constant amplitude loading.
- The retardation effect induced on fatigue crack growth rates by the application of overloads has been quantified from the calculation and analysis of the stress intensity factors defined in the CJP model.
- The size and shape of the crack tip plastic zone have been perfectly characterised by the use of the CJP model through their comparison with experimental results obtained by DIC.
- The results obtained in the present work try to contribute to a better understanding of the mechanisms driving fatigue crack propagation where plasticity plays a relevant role.

## Figures and Tables

**Table 1:** Mechanical properties for the 2024-T3 aluminium alloy and the commercially pure titanium tested in this work.

Mechanical Property	Unit	Value	
		AA2024-T3	CPTi
Young’s modulus	MPa	73,000	105,000
Yield stress	MPa	345	390
Poisson’s ratio	-	0.33	0.33

**Table 2:** Experimental conditions defined for the fatigue tests.

Investigated Aspect	Specimen Reference	Loading Conditions		Stress Ratio	Overload Conditions			
		*P_min_* (N)	*P_max_* (N)	*R*	Percentage (%)	*P_OL_* (N)	*a_OL_* (mm)	*N_OL_* (Cycles)
Crack shielding	AACT1	5	600	0	-	-	-	-
	AACT2	600	1200	0.5	-	-	-	-
Overload effects	AACT3	5	600	0	100	1200	26.1	280,000
	AACT4	5	600	0	125	1350	26.7	200,000
Crack tip plastic zone	TiCT1	450	750	0.6	-	-	-	-
	TiCT2	450	750	0.6	50	1125	6.19	77,000

## Data Availability

The data that support the findings of this study are available from the corresponding author upon reasonable request.
